# Quantitative analysis of the *Escherichia coli* proteome

**DOI:** 10.1016/j.dib.2014.08.004

**Published:** 2014-08-22

**Authors:** Jacek R. Wiśniewski, Dariusz Rakus

**Affiliations:** a Biochemical Proteomics Group, Department of Proteomics and Signal Transduction, Max-Planck-Institute of Biochemistry, Martinsried, Germany; b Department of Animal Molecular Physiology, Wroclaw University, Wroclaw, Poland

**Keywords:** Total protein approach, *Escherichia coli* proteome, Filter-aided sample preparation, FASP, Absolute protein quantification, Protein copy number

## Abstract

*Escherichia coli* (strain ATCC 25922 in a stationary culture) cells were lysed with SDS and the lysates were processed according MED-FASP protocol. The released peptides were analyzed by LC–MS/MS. Protein content per bacterial cell was calculated on the basis of the DNA content. Absolute protein quantitation was performed using the ‘Total Protein Approach’. The data are supplied in the article.

**Specifications table**

**Table:** 

Subject area	Biology, bacteriology
More specific subject area	Bacterial proteome
Type of data	Table, Figure
How data was acquired	Mass spectrometry using a Q Exactive mass spectrometer (Thermo Fisher Scientific, Germany)
Data format	Analyzed output data
Experimental factors	SDS lysates were processed using MED-FASP protocol
Experimental features	LysC and tryptic peptides were analyzed by means of LC-MS/MS
Data source location	Martinsried, Germany
Data accessibility	The data are with this article

**Value of the data**

• Quantitative picture of the *E. coli* proteome at protein copy number
• More than 2200 protein identified single per sample
• The protein abundances span 5 orders of magnitude

## 1 Experimental design

**Fig. 1:**
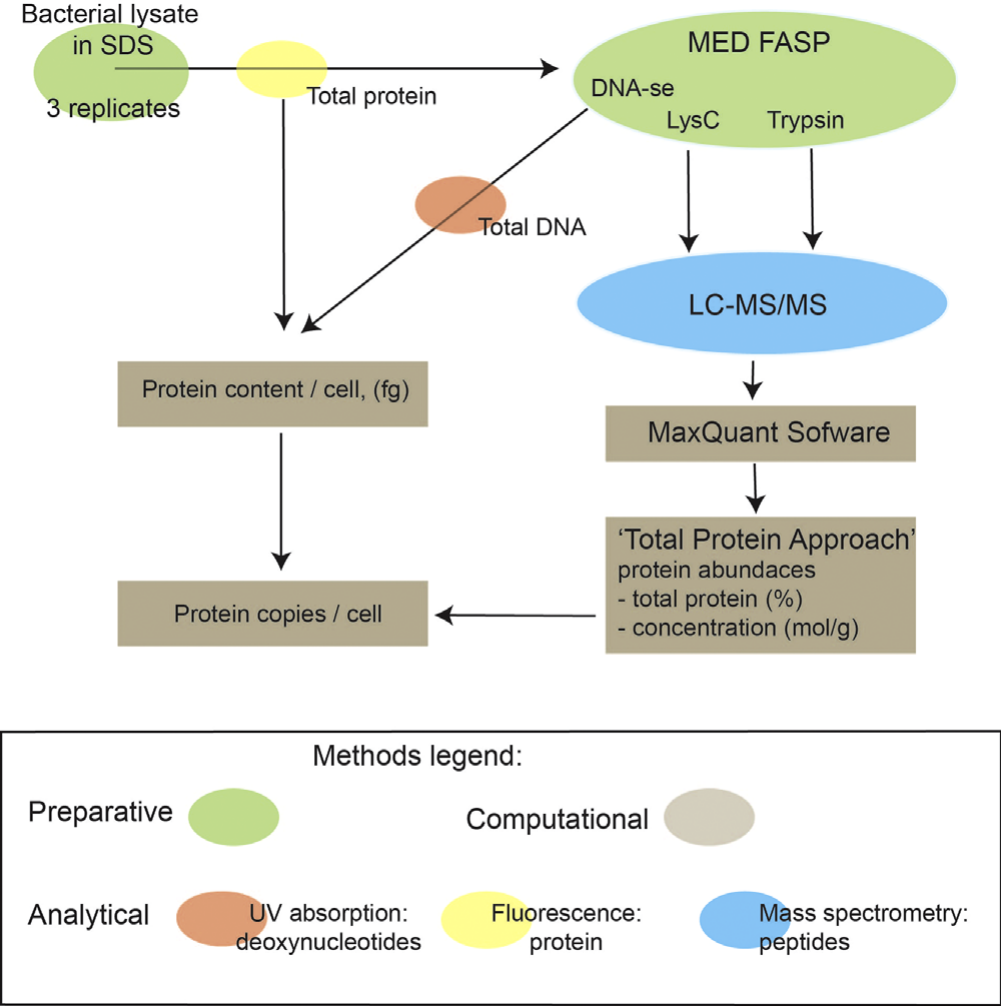
Schematic of the MED FASP protocol.

Bacterial lysates were processed according to MED FASP protocol (Fig. 1). Peptides were analyzed by LC–MS/MS and the resulting spectra were handled by the MaxQuant software. All peptides and proteins identified in this study are listed in Supplementary Tables S1 and S2 (for table legend see ‘Legends to Tables 1 and 2’), respectively. Absolute protein contents and protein concentrations were calculated using the total protein approach (Table 2). DNA was digested with nuclease and the released nucleotides were quantified. The total protein content of the single bacterial cell was calculated from the total DNA and total protein of the sample as described in [1]. The total protein content of the single cell was used for computation of protein copy numbers per cell (Table S2). Table S3 shows a selection of proteins involved energy metabolism in *Escherichia coli*.

## 2 Material and methods

### 2.1 Bacterial lysate

*E. coli* strain ATCC 25922 was cultured at 37 °C in Luria-Bertani broth medium with shaking at 250 rpm for approximately 15 h. The bacteria were harvested by centrifugation at 5000*g* and then lysed within 2% SDS in 0.1 M Tris–HCl pH 7.8 containing 0.1 M DTT at 100°C for 5 min. The non-soluble material was removed by centrifugation at 16,000*g*, and the supernatants were used for analysis.

### 2.2 Filter-aided protein and nucleic acid digestion

The lysates were processed according to the MED-FASP [2] protocol that was extended with nucleic acid digestion steps. Briefly, aliquots containing 50 μg total protein were mixed with 200 μL of 8 M urea in 0.1 M Tris/HCl, pH 8.5 [3] in centrifugal ultrafiltration units with a nominal molecular weight cut off of 30,000 (Cat no. MRCF0R030, Millipore), and then centrifuged at 14,000*g*, 20 °C, for 15 min. The eluates were discarded, 100 μL of UA was pipetted into the filtration unit, and the units were centrifuged again. Then 50 μL of 0.05 M iodoacetamide in UA was added to the filters, and samples were incubated in darkness for 20 min. Filters were washed twice with 100 μL of UA followed by two washes with 100 μL of 0.05 M Tris/HCl pH 8.5. Proteins were digested in 40 μL 0.05 M Tris/HCl pH 8.5 at 37 °C for 18 h, using endoproteinase LysC, at an enzyme to protein ratio of 1:50. The released peptides were collected by centrifugation at 14,000*g* for 10 min followed by two washes with 0.05 M Tris/HCl pH 8.5. After isolation of the peptides, material remaining on the filter was digested with trypsin using the above conditions, except that the cleavage reaction was performed for only 2 h. After collection of the peptides released by trypsin, the material remaining on the filter was washed once with TE buffer (10 mM Tris–HCl, pH 8.0) and then the RNA was digested with 0.5 μL (0.5U) of RiboShredder (Epicenter, Madison, WI) in 60 μL of TE at 37 °C for 1 h. The digested RNA was collected by centrifugation. Then the filters were washed twice with 80 μL of TE. Subsequently the filtration units were assembled in new tubes and the DNA was cleaved with 6 μg DNase (DN25, Sigma, St. Louis) in 60 μL of 10 mM Tris–HCl, pH 7.8 buffer containing 2.5 mM MgCl_2_ and 0.5 mM CaCl_2_. After 1 h incubation at 37 °C the DNA-digests were collected by centrifugation. The elution was completed by passing two 80 μL aliquots of the buffer.

### 2.3 Determination of the total protein and nucleic acid content

Total protein and total peptide content were determined using a tryptophan-fluorescence assay as described previously [4]. The DNA and RNA content was determined by UV spectrometry using the extinction coefficient of 0.025 (μg/mL)^−1^ cm^−1^ at 260 nm for ribonucleotides and 0.030 (μg/mL)^−1^ cm^−1^ at 260 nm for deoxyribonucleotides.

### 2.4 LC-MS/MS and data analysis

Aliquots containing 6 µg of LysC peptide or 4 µg of tryptic peptides were separated on a reverse phase column and analyzed on QExactive mass spectrometer as described previously [5]. The MS data were analyzed within the software environment MaxQuant [version 1.2.6.20] [6], using the Andromeda search engine (http://www.maxquant.org). Proteins were identified by searching MS and MS/MS data of peptides against UniProtKB Escherichia coli (K12) database. The FDR threshold was derived by analyzing the decoy database. Carboamidomethylation of cysteines was set as fixed modification. The maximum false peptide discovery rate was specified as 0.01. Spectra were searched with K-specificity for LysC and K/R but not K/RP for trypsin. Protein abundance was calculated on the basis of spectral protein intensity using the total protein approach (TPA) [7].

## Figures and Tables

**Table 1:** 

Sequence	Identified sequence
Missed cleavages (Lys-C)	Number of not cleaved sites by LysC
Missed cleavages (Trypsin/P)	Number of not cleaved sites by trypsin
Mass	Peptide mass
Proteins	UNIPROT ID
Gene names	Genes matching the peptide sequence
Protein names	Protein mathcing the peptide sequence
Charges	Ion charges
PEP	Posterior error probability
Score	MaxQuant peptide score
Intensity LysC sample 1	Spectral intesity of the peptide in LysC sample 1
Intensity LysC sample 2	Spectral intesity of the peptide in LysC sample 2
Intensity LysC sample 3	Spectral intesity of the peptide in LysC sample 3
Intensity trypsin sample 1	Spectral intesity of the peptide in tryptic sample 1
Intensity trypsin sample 2	Spectral intesity of the peptide in tryptic sample 2
Intensity trypsin sample 3	Spectral intesity of the peptide in tryptic sample 3

**Table 2:** 

Protein names	Protein names (UNIPROT)
Gene names	Gene names (UNIPROT)
Fasta headers	protein id FASTA header (UNIPROT)
Proteins	Number of proteins in the protein group
Peptides	Total number of identified peptides
Unique peptides	Number of identified unique peptides
Peptides sample 1	Total number of identified peptides in sample 1
Peptides sample 2	Total number of identified peptides in sample 2
Peptides sample 3	Total number of identified peptides in sample 3
Unique peptides sample 1	Number of identified unique peptides in sample 1
Unique peptides sample 2	Number of identified unique peptides in sample 2
Unique peptides sample 3	Number of identified unique peptides in sample 3
Sequence coverage [%]	Protein sequence coverage by all peptides
Unique sequence coverage [%]	Protein sequence coverage by unique peptides
PEP	Posterior error probability
Sequence coverage sample 1 [%]	Protein sequence coverage by all peptides identiffied in sample 1
Sequence coverage sample 2 [%]	Protein sequence coverage by all peptides identiffied in sample 2
Sequence coverage sample 3 [%]	Protein sequence coverage by all peptides identiffied in sample 3
Intensity sample 1	Summed spectral intesity of peptides matchin the protein id. In sample 1
Intensity sample 2	Summed spectral intesity of peptides matchin the protein id. In sample 2
Intensity sample 3	Summed spectral intesity of peptides matchin the protein id. In sample 3
Total intensity sample 1	Sum of spectral intesities of all peptides identified in sample 1
Total intensity sample 2	Sum of spectral intesities of all peptides identified in sample 2
Total intensity sample 3	Sum of spectral intesities of all peptides identified in sample 3
Total protein sample 1	Fraction of total protein in sample 1
Total protein sample 2	Fraction of total protein in sample 2
Total protein sample 3	Fraction of total protein in sample 3
Mol. weight [kDa]	Molecular weight protein
Mol. weight [kDa]	Molecular weight protein
Mol. weight [kDa]	Molecular weight protein
concentration pmol/mg sample 1	Protein concentration in sample 1
concentration pmol/mg sample 2	Protein concentration in sample 2
concentration pmol/mg sample 3	Protein concentration in sample 3
protein per cell (pg) sample 1	Total protein content per cell in sample 1
protein per cell (pg) sample 2	Total protein content per cell in sample 2
protein per cell (pg) sample 3	Total protein content per cell in sample 3
copy number per cell sample 1	Number of protein copies per cell in sample 1
copy number per cell sample 2	Number of protein copies per cell in sample 2
copy number per cell sample 3	Number of protein copies per cell in sample 3
Average copy number	Average sample 1-3
